# The Predictive Accuracy of the LSI-R in Female Forensic Inpatients—Assessing the Utility of Gender-Responsive Risk Factors

**DOI:** 10.3390/ijerph20054380

**Published:** 2023-03-01

**Authors:** Viviane Wolf, Juliane Mayer, Ivonne Steiner, Irina Franke, Verena Klein, Judith Streb, Manuela Dudeck

**Affiliations:** 1 Department of Psychiatry and Psychotherapy, Medical Faculty, LVR-Clinic Duesseldorf, Heinrich Heine University Duesseldorf, 40629 Duesseldorf, Germany; 2 Department of Forensic Psychiatry and Psychotherapy, kbo-Isar-Amper-Clinic Taufkirchen (Vils), 84416 Taufkirchen (Vils), Germany; 3 Psychiatric Services of Grisons, 7000 Chur, Switzerland; 4 Department of Forensic Psychiatry and Psychotherapy, Ulm University, 89312 Guenzburg, Germany

**Keywords:** female offenders, recidivism, mental illness, risk factors, gender, risk assessment

## Abstract

Female reoffending has long been a neglected research interest. Accordingly, risk assessment instruments were developed based on the criminological knowledge of male recidivism. While feminist researchers have repeatedly criticized the failure to incorporate gender-responsive risk (GR) factors, opinions on the gender neutrality of existing instruments remain inconsistent. In order to substitute the existing literature, while extending the scope to mentally disordered offenders, the aim of the given study was the prediction of general recidivism in a sample of 525 female forensic inpatients who had been discharged from forensic psychiatric care in Germany between 2001 and 2018. Primarily, ROC analysis was conducted to assess the predictive accuracy of the LSI-R. Subsequently, separate binary logistic regression analyses were performed to determine the predictive utility of GR factors on recidivism. Lastly, multiple binary logistic regression was used to assess the incremental validity of the GR factors. The results showed that the GR factors (i.e., intimate relationship dysfunction, mental health issues, parental stress, adult physical abuse, and poverty) significantly contributed to the prediction of recidivism, while a mixed personality disorder, a dissocial personality, an unsupportive partner, and poverty added incremental validity to the predictive accuracy of the LSI-R. However, given that the added variables could only improve classification accuracy by 2.2%, the inclusion of gender-specific factors should be cautiously evaluated.

## 1. Introduction

Due to proportionally low incarceration rates, female criminality has long been an underrepresented research interest [1,2]. Within recent decades, however, rising incarceration rates have been recorded for female offenders globally, whilst the opposite trend was indicated for male offenders [3,4]. The recognition of the narrowing gender gap noticeably increased scientific attention directed to female offending. Consequently, gender differences in the risk factors underlying the initiation and persistence of offending, as well as desistance from criminal behavior, have been repeatedly highlighted [5,6]. Centrally, these factors include experiences of abuse, economic marginalization, parental stress, mental health, and intimate relationship dysfunction [1,7,8,9]. Most clearly, with respect to the prediction of future offending, individual risk and need profiles are essential in choosing adequate offender treatment, while reducing the risk to public safety. In order to unify this process, various risk assessment instruments have been developed in recent decades. As the prevailing model of offender rehabilitation, the risk–need–responsivity (RNR) model [10], also referred to as the Canadian model [11], forms the basis for common dynamic risk assessment tools. When applied to various offending populations, including mentally disordered offenders, the model has proven its suitability and has thus become widely accepted [12]. According to the application of this model, the intensity and textual focus of offender treatment needs to be adapted to an individual risk and need profile—one that is determined by a collection of factors that are predictive of future offending. However, while drawing on numerous studies concerning male reoffending, researchers have repeatedly failed to integrate the knowledge of female offenders. Consequently, the risk assessment tools that emerged within the last few decades are almost entirely based on the criminological knowledge of male offenders [6,13]. Grounded in the assumption that male and female offenders display comparable risk factors, these instruments have been regularly applied to female offenders. This assumption, however, not only neglects findings on gender-responsive (GR) factors, but also underestimates the uniqueness of the female offender population [14,15]. In fact, male and female offenders display substantial differences with regard to the nature of offending, as well as the underlying motivations. Essentially, women offend at a lower frequency, with fewer violent crimes and with more drug- and property-related offenses relative to their male counterparts [15,16]. In addition, violent offending by women is more often considered reactive and relational, while male violence is more frequently regarded as instrumental and driven by status or financial gain. Equally, women’s crimes tend to be motivated by feelings of frustration or jealousy, while men are more likely to have selfish or antisocial motivations [17]. Additionally, gender differences appear in regard to the responsivity to supervision and treatment options [16], as well as classification accuracy, thereby resulting in harsher treatments for females than may be appropriate [18,19].

Building on these weaknesses, the suitability of existing instruments in female offender populations has repeatedly been questioned [1,6]. In parallel, feminist researchers have been stressing the need for gender-specific assessments [20,21]. At present, this has resulted in two female-specific instruments being developed and increasingly used in current clinical practice. In regard to the prediction of violent reoffending, the Female Additional Manual (FAM) [22] was designed as a gender-responsive supplement to the Historical Clinical Risk Management-20, Version 3 (HCR-20 V3) [23]. For the purposes of predicting general recidivism, the women’s risk needs assessment (WRNA) [21] was developed to account for female risk/need domains. This assessment is available as a stand-alone version and as a supplement to the Level of Service Inventory-Revised (LSI-R) [24]. Currently, little empirical evidence exists evaluating the predictive accuracy of these instruments. Yet, two studies have validated the incremental validity of the WRNA in different countries [11,25]. The first results of the FAM could only confirm the clinical relevance of the instrument, without providing incremental validity to the HCR-20 V3 [26].

Proponents of the Canadian model, however, disagree with feminist criticism by claiming the gender neutrality of current dynamic risk instruments [11]. Particularly, they argue that the RNR principles—which form the basis of the LSI-R [24]—are based on the general personality and cognitive social learning (GPCSL) theory of crime [10]. This theory entails the understanding of criminal behavior as the result of cost/benefit considerations, which are supposedly unaffected by gender [27]. Respectively, Bonta et al. [10] claim that the included risk factors—which centrally concern “criminal history, antisocial personality patterns, antisocial associates, education, and substance abuse” should be interpreted as “general” rather than male specific. In support of this perspective, the LSI-R is currently considered the most promising candidate in terms of gender neutrality [28]. Building on the repeated confirmative results in regard to the suitability of the LSI-R for female offenders [27,29,30,31], a meta-analysis of 14,737 female offenders [32] was conducted. Respective researchers concluded that the performance of the LSI-R is comparable for both genders.

Notwithstanding these encouraging findings, feminist scholars emphasize that even the most recent risk assessment instruments were primarily developed for male offenders and only later applied to female offenders. Likewise, the relevance of the included risk factors to female offenders has not been reviewed, while delayed attention was directed to the validity testing. Equally, findings on the validity were lacking consideration of the gender-responsive literature. Consequently, it remains to be clarified if instruments that are specific to women can further improve recidivism prediction in female offender populations. While some studies confirm that the prediction accuracy of the LSI-R improves by adding gender-responsive factors [11,33], others have concluded the opposite [27]. As an attempt to explain contradicting findings, Reisig et al. [34] suggested that the LSI-R is only predictive for female offenders who did not follow gendered paths into crime. In order to gradually approach an answer to this ongoing discussion, the need to further substantiate findings on the applicability of the LSI-R in female offenders is clearly recognizable. Further, it is necessary to verify the generalizability of the existing evidence by studying various offending populations. Given that research on this topic exclusively addresses prison populations, studies on forensic populations are sorely needed. Specifically, with regard to the incremental validity of gender-responsive factors, when applied in conjunction with the LSI-R, evidence on the population of female forensic inpatients is urgently required. Recent research stresses this need by showing a strong overrepresentation of women in German forensic care relative to the proportion of women in prison. Equally, a strong increase in the number of female inpatients treated in German forensic facilities was found over the last few decades [35].

According to Van Voorhis et al. [11], gender-responsive factors are defined as “not typically seen in men, seen in men but occur at greater frequency in women, or occur in equal frequency among men and women but affect women in uniquely personal and social ways that should be reflected in current correctional assessment.” When reviewing the existing literature on risk/need factors of female recidivism, the following domains appear to be unique for female offenders [11,33]:

### 1.1. Mental Health

Research has consistently shown that female offenders fundamentally differ from male offenders in terms of mental health needs [11]. Compared to their male counterparts, women who come into contact with the criminal justice system (justice-involved women) are more frequently diagnosed with depression, anxiety/panic, self-harming behaviors, eating disorders, bipolar disorder, post-traumatic stress disorder (PTSD), and co-occurring substance abuse disorders [11,16,36]. Further, female offenders more often suffer from personality disorders compared to male offenders or the general population [2,37]. With respect to reoffending, various mental health issues—including emotional well-being [38], stress, depression, fearfulness, suicidality, and self-harm—were found to be predictive [8,11]. Correspondingly, desistance from criminal behavior is associated with stability in patients’ mental health [39]. While some risk assessments do capture the presence of a mental disorder, they mostly fail to distinguish between the various possible diagnoses [14].

### 1.2. Trauma and Abuse

Physical and sexual abuse are believed to play a critical role in the development of delinquent behavior [40]. In accordance with this assumption, female offenders report experiences of abuse significantly more often (up to 80%) than the general population [16,41]. Additionally, justice-involved women are experiencing abuse more frequently than their male counterparts [42]. Further, a significant amount of research has proven the influence of trauma and abuse on female recidivism, including general abuse, as well as childhood trauma/abuse and adult victimization [8,11,33].

### 1.3. Self-Efficacy and Self-Esteem

Self-esteem and self-efficacy are believed to be directly linked to female “empowerment” [11]. Confidence in their own abilities to achieve their goals is assumed to be a crucial factor for the process of desistance in female offenders [43]. With regard to recidivism specifically, however, there has been little research that has investigated the influence of self-esteem/self-efficacy. Having said this, one study has yielded promising findings, thereby showing the predictive ability of low self-esteem in regard to recidivism [11].

### 1.4. Intimate Relationships

Given that female offenders frequently report experiences of abuse, which in turn limits their ability to comply with trusting and healthy relationships, it is not surprising that female offenders regularly enter dysfunctional relationships [44]. A study on female forensic patients revealed that up to 2/3 of the patients reported an unstable intimate relationship [2]. Regarding recidivism, evidence has shown that relational deficits represent a major risk factor in females. In comparison to male offenders, the relationships of justice-involved women appear to be directly linked to their probabilities of reoffending [44,45]. Risk factors, particularly, cover dysfunctional intimate relationships, criminal partners [8], and a higher number of sex partners [46]. In contrast, when women reported good-quality intimate relationships, the recidivism risk declined [47].

### 1.5. Motherhood and Parental Stress

The importance of social bonds not only accounts for intimate partner relationships, but also familial relationships, including maternal bonds to children. It was found that losing custody or being separated from their children was significantly more stressful and onerous for mothers in comparison to fathers [44]. Further, research on recidivism confirmed the relevance of factors related to motherhood. In particular, parental stress [11], the loss of child custody, as well as out-of-home placement [48] were all proven to predict recidivism. Motherhood, on the other hand, was shown to be a protective factor for reoffending [49]. 

In addition, the following domains were found predictive of both genders, but assumed to affect females differently [33,41].

### 1.6. Addiction

Research has shown that female offenders suffer from substance use disorders at significantly higher rates than male offenders [50]. For women with mental disorders and histories of abuse, the rates are even higher [51]. Further, substance abuse more often serves as a coping mechanism used to deal with traumatic experiences and mental health issues in female offenders compared to their male counterparts [51]. When assessing recidivism, drug use was found to be more predictive in female offenders relative to male offenders [8]. With regard to desistance, refraining from drug use increased the probability of moving away from criminality for justice-involved women [39].

### 1.7. Economic Marginalization/Poverty

As highlighted by the prior literature, the biggest proportion of female offenders is suffering from poverty or financial needs prior to their incarceration [11,41]. Further, poverty was found to significantly increase the odds of recidivism in female offenders [52]. Moreover, it was shown to be associated with prostitution and further illegal activity [53]. Financial independence, correspondingly, was associated with the process of desistance from crime [39].

### 1.8. The Present Research

The aim of this study was to validate the applicability of the LSI-R in the context of mentally disordered female offenders. To our knowledge, no previous study has assessed the instrument in a female sample of forensic inpatients. In addition, we aimed to clarify whether gender-responsive risk factors are suitable to improve the predictive accuracy of the LSI-R in female forensic inpatients, which also has not been previously tested in the context of this population. Apart from these specific aims, we attempted to replicate prior findings on the existence of gender-responsive risk factors in the case of female recidivism and substitute literature on the feasibility of the LSI-R in female offenders.

## 2. Materials and Methods

### 2.1. Sample Characteristics

The study sample comprised 525 female forensic inpatients who had been discharged from a forensic psychiatric hospital in Germany (Bavaria) between 2001 and 2018. Each woman had received court-ordered treatment, either according to section 63 or section 64 of the German Penal Code. Admission by section 63 requires the diagnosis of a severe mental illness that is decisive for the committed offense; diminished criminal responsibility; and a significant risk of reoffending. The treatment duration is not limited a priori but it is reviewed annually. Admission by section 64, on the other hand, requires the diagnosis of a substance use disorder that is centrally linked to the initial offense; a significant risk of reoffending; and a positive treatment prognosis. The treatment duration, in general, is at least 2 years but can vary according to additional prison sentences. In contrast with section 63, patients admitted by section 64 can be discharged if the requirements for successful treatment completion are not being fulfilled anymore and, depending on concurrent prison sentences, may return to prison. For the purposes of the current study, the inclusion criteria were a minimum age of 18 at the time of discharge and a final conviction for placement in a forensic hospital. In addition, only those cases for which sufficiently informative data were available were included. In the final study sample, the proportion of missing data was very limited. Across the included variables, between 0% to 5% of the data were missing. Additionally, as missing values were randomly distributed (MCAR), any cases with missing data on any of the variables of interest were excluded from the analyses and no missing data were imputed.

The mean age at admission was 34.1 years (range: 16–67 years). The mean duration of inpatient treatment was 32.02 months (range: 0–152 months). The mean follow-up period was 8.98 years (2.05–19.68 years). The recidivism rate was 39.6%. Concerning the diagnoses of the given sample, 72.1% were diagnosed with a substance use disorder (ICD-10, F1), of which 22.7% were given the diagnosis of an alcohol use disorder. Further, 18.5% of the sample were diagnosed with a psychotic disorder (ICD-10, F2), while 4.4% were given the diagnosis of an affective disorder (ICD-10, F3) and 3.4% were diagnosed with a neurotic disorder (ICD-10, F4). Lastly, 20.6% were diagnosed with a personality disorder (ICD-10, F6), of which 4.8% were diagnosed with a mixed personality disorder. A total of 33.5% were rated emotionally unstable (i.e., accentuation of traits/personality disorder), while 16.8% were provided with a diagnosis of an emotionally unstable personality disorder. Likewise, 22.1% were rated dissocial (i.e., accentuation of traits/personality disorder), while only 2.5% were diagnosed with a dissocial personality disorder. With regard to the index offenses, 43.4% of the sample had committed a violent offense—of which 5.5% were convicted of arson, 5.5% of robbery, 8.8% of homicide, and 21.3% of bodily harm. Among the non-violent offenders, 38.3% were convicted for a drug-related offense while 14.3% had committed a property offense.

### 2.2. Materials

#### 2.2.1. The Level of Service Inventory-Revised (LSI-R)

The LSI-R [24] is a standardized, actuarial risk assessment instrument. It is designed to assess the risk of general recidivism and to correspondingly suggest adequate treatment and risk management options. It consists of 54 risk factors, all of which are considered to be the most predictive of future offending. The instrument is divided into 10 contextual domains, including: criminal history; occupational and financial aspects; factors related to family; intimate partners and friends; housing conditions; recreational activities; alcohol and drug problems; emotional and personal impairment; and lastly, personal attitudes and values. In respect to the given study, the German version of the LSI-R was used. The interrater reliability of the instrument was considered high (r = 0.80 to r = 0.90), while the internal consistency was reported moderate to high (r = 0.41 to r = 0.69) [54]. Additionally, the predictive validity of the original version was considered good (r = 0.35 to r = 0.38) [55].

#### 2.2.2. The Female Additional Manual (FAM)

The FAM is a gender-responsive violence risk assessment instrument, which was developed to supplement the HCR-20 V3 with GR factors and additional guidelines adjusted to female offenders. As it was constructed to predict violent recidivism, the instrument was not suitable for application to the present study. However, as some of the instruments’ risk factors overlap with the GR risk factors of general recidivism, the item definitions that corresponded with out intended specificity were used in the given study. Particularly, this concerned the items “low self-esteem” and “problematic childcare responsibility”, the latter being defined as “the heavy burden and responsibility of taking care of an underage child or children” and/or “the anger, frustration and sorrow that can result from the loss of contact or limited contact with children” [22].

### 2.3. Procedure

The present study was part of a larger project regarding the applicability of common risk assessment instruments in female forensic inpatients. In collaboration with the Office of Corrections and Rehabilitation, Zurich, Switzerland, a codebook was designed. Provided with the item definitions and the respective rating scheme, the codebook served as a detailed coding guide for the included items (i.e., sociodemographic data, gender-responsive risk factors and risk assessment instruments). Following a detailed literature review, gender-specific risk factors with sufficient empirical evidence were selected. Contextually, these factors concerned the following variable domains: mental health (e.g., diagnoses); trauma/victimization (e.g., experiences of sexual violence during childhood); intimate partner dysfunction (e.g., unstable intimate relationship); parental stress (e.g., loss of child custody); self-esteem (low self-esteem); and poverty (e.g., homelessness). Diagnoses were coded according to the ICD-10 criteria. The item definitions were mainly created based on the available literature. For those factors adopted from the FAM [22], we used the corresponding definitions of the instrument. All gender-responsive factors were dichotomously coded (yes/no). To counteract multicollinearity, we calculated the correlations between the gender-responsive risk factors and the LSI-R items using Chi square tests of independence. Substance abuse, although labeled gender responsive, was not assessed separately, as it is already included in the LSI-R. For the purposes of coding the risk assessment instruments, the five raters received professional training to ensure the correct application. In order to verify the agreement of the raters, interrater reliability testing was carried out on the risk assessment instruments, which delivered good results for the LSI-R (ICC = 0.735*, CI = 0.483; 0.919). Next, the Codebook was rated based on file information, i.e., archived patient records, including official court documents. As patient files differed in quality and completeness, it was specified beforehand to only include those files that provided sufficient information to accurately assess the Codebook. In order to be considered for the study, it was required that at least the initial court decision/the initial forensic psychiatric assessment and a final report on the therapeutic process be available.

In order to assess general recidivism, extracts from the Federal Central Criminal Register were requested in September 2020 and February 2021. Any reconviction was coded as recidivism with a binary measure (yes/no). To ensure the estimates between the risk factors and recidivism were not biased, we included three control variables. First, the patients’ age was included, measured in years at the time of admission to forensic care. Second, the time at risk was used, reflecting the years between discharge from the forensic psychiatric facility and the date the criminal register was extracted. Third, the duration of treatment was included, mirroring the months between the admission date and the date of discharge. All three control variables were measured on a continuous scale.

Data collection took place between 2019 and 2021. The study was approved by the Ethics Committee of the Bavarian Medical Association (approval no. 2019-167).

### 2.4. Statistical Analyses

Analyses were conducted using IBM SPSS Statistics Version 29 [56]. Primarily, receiver operating characteristic (ROC) analysis was conducted to determine the predictive validity of the LSI-R in regard to general recidivism after discharge. In order to simplify comparisons across different studies, research on risk assessment commonly uses ROC analysis [26]. According to the guidelines of Rice and Harris [57], AUC values between 0.56 and 0.64 are considered a low effect (Cohen’s *d* 0.20), values between 0.64 and 0.71 are a medium effect (Cohen’s *d* 0.50), and values of 0.71 or above a high effect (Cohen’s *d* 0.80). For the subsequent analyses, the overarching goal was to determine the incremental validity of the gender-responsive factors. For this purpose, we started conducting separate multiple binary logistic regressions to determine the GR factors that significantly contributed to the prediction of recidivism, each being controlled for age, treatment duration, and time at risk. Subsequently, another series of multiple binary logistic regression analyses was conducted to clarify which GR factors uniquely predicted recidivism, net the variance explained by the LSI-R sum score and the control variables. In preparation for the actual analysis of interest, all GR factors that were found to be significant at the previous stage were inserted into multiple binary logistic regression analysis while controlling for the LSI-R sum score and the controls. Using the backward elimination method, the GR factors that uniquely contributed to the model performance were determined and selected for subsequent analysis. We then proceeded to the logistic regression of interest, which was carried out in three steps. In the first step, the three control variables were inserted. In the second step, the LSI-R sum score was entered and in the last step, all gender-responsive variables were added. In order to calculate the incremental validity of model three (i.e., controls, LSI-R sum score and GR factors) over model two (i.e., controls and LSI-R sum score), we evaluated the goodness of fit for both models using Chi-square testing, with a significance threshold of 0.05.

## 3. Results

### 3.1. Predictive Accuracy of the LSI-R

Receiver operating characteristic (ROC) analysis was conducted on the total sample (N = 525) in order to determine the predictive accuracy of the LSI-R. As shown in Table 1, the AUC (area under the curve) was statistically significant when predicting recidivism. An AUC value of 0.748 indicates a high effect (AUC > 0.71).

### 3.2. Predictive Utility of the Gender-Responsive Factors on Recidivism

Multiple binary logistic regression was performed to predict recidivism using the gender-responsive risk factors. Each GR factor was assessed in a separate regression model, controlling for age, treatment duration, and time at risk. As displayed in Table 2, several gender-responsive risk factors significantly contributed to the prediction of recidivism. First, mental health issues were strongly related to an increased likelihood of reoffending. Being diagnosed with a mixed personality disorder nearly quintupled the odds of recidivism, while a dissocial personality (i.e., accentuation of traits/disorder) nearly tripled the probability of reoffending. Equally, the odds of recidivism increased for those patients that reported physical violence during adulthood. Further, a heightened probability of recidivism was associated with intimate relationship dysfunction and risky sexual behavior. While having an unstable intimate relationship at the time of discharge from the forensic facility nearly tripled the odds of recidivism, an unsupportive partner doubled the probability of reoffending. A history of high-risk sexual behaviors as well as a higher number of sexual partners increased the odds of recidivism. In addition, parenting difficulties were associated with higher probabilities of reoffending. While the loss of child custody prior to admission to forensic treatment doubled the odds of recidivism, problematic childcare responsibility at the time of discharge from forensic care nearly doubled the odds. Lastly, indicators of economic marginalization strongly correlated with the odds of reoffending. While a history of poverty tripled the odds of reoffending, experiences of homelessness increased the odds of recidivism. On the contrary, experiences of childhood abuse (i.e., sexual, emotional, physical), adult sexual and emotional abuse, as well as affective (ICD-10, F3) and neurotic disorders (ICD-10, F4), an emotionally unstable personality (i.e., accentuation of traits/disorder), previous self-harm, previous suicide attempts, and low self-esteem failed to contribute to the prediction of recidivism.

### 3.3. Incremental Validity of the Gender-Responsive Risk Factors

Multiple binary logistic regression was performed in three steps in order to determine whether the addition of gender-sensitive variables improved the predictive accuracy of the LSI-R. In the first step, the selected control variables (i.e., age, treatment duration and time at risk) were inserted. In the second step, the LSI-R sum score was added and in the third and final step, the GR factors were entered. In preparation for the analysis of interest, the suitable GR predictors were selected. For this purpose, all significant GR factors, the control variables, and the LSI-R sum score were inserted into multiple binary logistic regression analysis. Using the backward elimination method, the predictors that uniquely contributed to the model performance were determined. Consequently, the following GR factors were selected for the subsequent analysis: mixed personality disorder, dissocial personality (i.e., accentuation of traits/disorder), unsupportive partner, and poverty. As proportions of missing values among the selected variables were very minor, only four subjects had to be excluded from the analyses. Hence, the sample size for the final multiple binary logistic regression was 521. As displayed in Table 3, the first step of the multiple logistic regression, which included the selected control variables only, was found to be statistically significant (*x^2^* = 58.275, *df* = 3, *p* < 0.001). The model explained 14.3% (Nagelkerke *R^2^* = 0.143) of the variance in recidivism and correctly classified 65.8% of the cases. The recidivists were correctly classified by 44.0% and the non-recidivists by 80.3%. The odds of recidivism increased by 5% with every additional year at risk. The odds of reoffending decreased with higher age and longer treatment duration.

As shown in Table 4, the model in step two of the multiple logistic regression, which additionally included the LSI-R sum score, significantly improved the goodness of fit of the model (*x^2^* = 118.334, *df* = 4, *p* < 0.001). Equally, the classification accuracy increased by 4.8% and the explained variance increased by 13.2%. The model explained 27.5% (Nagelkerke *R^2^* = 0.275) of the variance in recidivism and correctly classified 70.2% of the cases. Recidivists were correctly classified by 56.0% and non-recidivists by 79.6%. The odds of recidivism increased by 11% for each rising unit on the LSI-R sum score. After entering the LSI-R sum score into the model, age was no longer significant.

As displayed in Table 5, the model in step three of the multiple logistic regression, which further contained the gender-responsive factors, significantly improved the goodness of fit for the model (*x^2^* = 141.073, *df* = 8, *p* < 0.001). Likewise, the classification accuracy improved by 2.2% and the explained variance increased by 4.6% relative to step two. Consequently, 14 patients who had been misclassified in step two were now classified correctly. The final model explained 32.1% (Nagelkerke *R^2^* = 0.321) of the variance in recidivism and correctly classified 72.4% of the cases. The recidivists were correctly classified by 58.0% and the non-recidivists by 81.8%. With regard to the included GR factors, the diagnosis of a mixed personality disorder, an unsupportive intimate partner, and poverty reached significance at *p* < 0.05 and contributed to an improved model performance. The diagnosis of a dissocial personality and the control variables age were not found to be significant at *p* < 0.5 but improved the performance of the model.

## 4. Discussion

Primarily, the goal of the current study was to verify the applicability of the LSI-R in predicting recidivism in female forensic psychiatric inpatients. Additionally, it aimed to investigate whether the supplementary assessment of gender-responsive risk factors provides incremental validity to the prediction of reoffending in mentally disordered female offenders using the LSI-R. Further, this article serves to verify existing findings on the feasibility of the LSI-R in female offenders. By using a sample of female forensic inpatients, we also contribute to the knowledge on risk factors for reoffending in regard to a highly understudied group of female offenders. The results indicate that the LSI-R is applicable for accurately predicting recidivism in female forensic inpatients. Specifically, the ROC analysis revealed a good predictive accuracy for the instrument. This finding is in line with previous findings in regard to female offender populations, thereby confirming the accurate predictive ability of the LSI-R for general recidivism [11,27,29]. Consequently, the current article provides evidence of the feasibility of the LSI-R in regard to mentally ill female offenders, even if the inferences on gender neutrality need to be interpreted cautiously without a comparison group of male offenders. Additionally, the results presented in this article show the importance of gender-responsive risk factors in the context of female recidivism. Specifically, various gender-responsive factors were found to be significant predictors of recidivism, even after controlling for the effects of age, treatment duration, and time at risk. In particular, measures of parental stress (i.e., loss of child custody and problematic childcare responsibility), intimate relationship dysfunction (i.e., unstable and unsupportive intimate relationships, higher sex partners, and high-risk sexual behaviors), economic marginalization (i.e., poverty and homelessness), mental health issues (i.e., dissocial personality and mixed personality disorder), and experiences of violence during adulthood were significantly associated with higher probabilities of recidivism. These findings are in line with previous studies confirming the predictive utility of gender-specific risk factors on female recidivism [11,32,44,45,46]. Further, this study supports feminist claims that stress the relevance of capturing those factors when assessing recidivism risk and applying adequate offender treatment. 

However, the mentioned analyses also generated findings that did not correspond with the prior literature on gender-responsive factors. Specifically, the domain of trauma produced conflicting results. While physical abuse during adulthood significantly predicted recidivism, the remaining measures of abuse and trauma were not associated with recidivism in the current study. This finding contradicts various earlier findings on female recidivism, which showed a significant correlation between childhood trauma and reoffending [8,11]. However, it replicates other findings negating this relationship [42]. A possible explanation may be the base rate of childhood trauma in the given sample. In fact, 72% of the study sample reported some sort of traumatic experience during childhood. In turn, the current article appears to confirm the negative effect of childhood trauma on female delinquency, but not on reoffending in particular. Further, findings concerning the domain of mental health have not uniformly replicated prior findings. In particular, current findings could not validate the increased recidivism risk of female offenders with affective and neurotic diagnoses [8,11]. In fact, these diagnoses were not able to predict recidivism in the given sample. Additionally, we generated confirmative findings on the negative effect of mixed personality disorders in regard to recidivism that had not been found previously [58]. At present, however, little research has addressed the influence of mixed personality disorders on recidivism in female offenders.

Concerning the second aim of the given article, the findings highlight the incremental validity of gender-responsive risk factors when added to the LSI-R. In specific, the results of the multiple binary logistic regression showed that the additional assessment of gender-specific risk factors significantly improved the overall performance of the model while controlling for age, treatment duration, time at risk, and the LSI-R sum score. This finding suggests that female-specific factors present unique predictive value that is not covered by the factors included in the LSI-R. Specifically, the highest predictive accuracy was obtained by adding the following gender-specific variables: mixed personality disorder; dissocial personality; unsupportive intimate partner; and poverty. Being diagnosed with a mixed personality disorder was associated with four times greater probability of recidivism. As mentioned earlier, these findings contradict earlier studies that did not find a correlation between mixed personality disorders and recidivism [58]. However, the overwhelming majority of the findings validated the prior literature on gender-responsive risk factors. Primarily, the heightened recidivism risk that arises from problematic intimate partner relationships could be confirmed, which specifically applied to the support provided by a partner [11,33,47]. Secondly, we found additional support for the unique effect of mental health on female recidivism [8,11,38,39]. In particular, it was shown that a diagnosis of dissocial personality disorder/accentuation of traits contributed to increased predictive accuracy. This finding corresponds with prior findings that highlighted the critical role of antisocial personality characteristics in female recidivism [59]. Moreover, the current findings could support earlier studies on the negative effect of poverty in regard to recidivism [52]. Particularly, experiences of poverty doubled the probability of recidivism. Overall, the current findings verify the unique predictive value of gender-responsive predictors on female reoffending. Consequently, attending to these factors constitutes a useful addition to the risk assessment and treatment planning of female offenders. In light of the significant, but rather limited, improvement in prediction accuracy, the inclusion of gender-responsive risk and need factors may have stronger implications for clinical practice than for risk assessment. For clinical purposes, it appears strongly recommended to incorporate gender-responsive risk/need factors in order to provide treatment options that are best suited to this specific population.

### Limitations, Strengths, and Future Directions

The current study has certain limitations that should be addressed in future research. First, the retrospective, file-based assessment of data results in several shortcomings. In particular, patient records—which mostly concerned professional forensic documentation—differed in content. While some records described the investigated variables in detail, others covered them only superficially. Further, we were not able to apply the LSI-R as originally intended (i.e., in interview form). The results from the logistic regression, however, revealed comparable levels of predictive accuracy relative to studies using interview forms. It can therefore be assumed that the retrospective assessment of the LSI-R in the current study did not interfere with the study’s aim. For future research, it would be advised to verify the obtained results in a prospective study using an interview form. Second, due to the nature of this study, we could only use external measures, neither capturing the subjective view of the patients nor verifying file information. By using a mixed-methods design, future research could address this limitation. Third, for the measure of recidivism, we exclusively relied on official records of re-convictions and did not consider any other measures of repeated offending. Future studies could also include arrests or self-reported reoffending. Lastly, the used sample included female offenders only. Without a comparison group of male offenders, conclusions on gender-specific risk factors need to be interpreted with caution. While addressing this limitation with comparisons to the existing literature, comparative gender studies are required in order to draw more reliable inferences.

Despite the limitations, the current study contributes substantially to the existing literature on the risk assessment of female offenders. Further, this article addresses a common methodological flaw in this field by providing a good sample size (N = 525) from which to draw reliable conclusions. Additionally, the given article provides urgently needed evidence on the highly understudied population of female forensic inpatients. To our knowledge, this study currently has the highest sample size for studies on recidivism in female forensic inpatients in German forensic care. Consequently, inferences from this article may assist in improving the risk assessment and treatment planning for females in forensic treatment in Germany.

## 5. Conclusions

The current article provides support for the suitability of the LSI-R in female forensic inpatients. Similarly, the findings highlight the importance of an additional assessment of gender-responsive risk factors with regard to the risk assessment and treatment planning of mentally disordered female offenders. In particular, the presented results revealed significant gender-responsive predictors of recidivism, including a dysfunctional intimate relationship, mental health issues (i.e., dissocial personality and mixed personality disorder), parental stress, adult physical abuse, and poverty. Further, unique predictive value was found for the following factors: dissocial personality (i.e., accentuation of traits/disorder), mixed personality disorder, unsupportive intimate relationship, and poverty, even after controlling for age, treatment duration, time at risk, and the LSI-R sum score. Additionally, the inclusion of the gender-responsive risk factors contributed to improvements in the predictive accuracy of the LSI-R. Clinical practice may be advised to attend to gender-responsive risk/need factors in order to enhance the chances of successful treatment in female offenders. Further research is required to validate the given findings, preferably using prospective, mixed study designs, as well as by including a male comparison group.

## Figures and Tables

**Table 1:** Results of the AUC analysis used to assess the predictive accuracy of the LSI-R.

	AUC	CI 95%	Standard Error	*p*
LSI-R	0.748	(0.707, 0.790)	0.021	<0.001

**Table 2:** Multiple binary logistic regression analyses predicting recidivism based on gender-responsive risk factors.

	Exp (b)	Significance
Emotionally unstable personality (F60.3, F61.0, and Z73.1)	1.414	0.089
Dissocial personality (F60.2, F61.0, and Z73.1)	2.735	<0.001
Mixed personality disorder (F61.0)	4.950	<0.001
Affective disorder (F3)	1.221	0.663
Neurotic disorder (F4)	1.988	0.177
Previous suicide attempts	1.406	0.090
Previous self-harm	1.396	0.125
Physical violence during childhood	1.330	0.142
Emotional violence during childhood	0.945	0.769
Sexual violence during childhood	1.409	0.093
Physical violence during adulthood	1.510	0.034
Emotional violence during adulthood	1.032	0.871
Sexual violence during adulthood	1.070	0.771
Unstable intimate relationship	2.841	<0.001
Unsupportive intimate partner	2.222	<0.001
A higher number of sexual partners	1.171	0.006
High-risk sexual behavior	1.704	0.009
Loss of child custody	2.036	0.001
Problematic childcare responsibility	1.719	0.010
Poverty	3.059	<0.001
Homelessness	1.626	0.025
Low self-esteem	1.109	0.592

Regressions were controlled for age, treatment duration and time at risk.

**Table 3:** Step 1: Multiple binary logistic regression predicting recidivism based on the control variables age, treatment duration and time at risk (N = 521).

	Exp (b)	Significance
Age	0.967	<0.001
Treatment duration	0.974	<0.001
Time at risk	1.052	0.015

**Table 4:** Step 2: Multiple binary logistic regression predicting recidivism based on the LSI-R sum score and the control variables (N = 521).

	Exp (b)	Significance
LSI-R	1.110	<0.001
Age	0.985	0.159
Treatment duration	0.988	0.011
Time at risk	1.062	0.007

**Table 5:** Step 3: Multiple binary logistic regression predicting recidivism based on the LSI-R sum score, the control variables, and the GR factors (*N* = 521).

	Exp (b)	Significance (p)
LSI-R	1.077	<0.001
Age	0.989	0.317
Treatment duration	0.983	0.002
Time at risk	1.073	0.003
Dissocial (F60.2, F61.0, and Z73.1)	1.630	0.056
Mixed personality disorder (F61.0)	3.861	0.007
Unsupportive intimate partner	1.787	0.007
Poverty	2.065	0.017

## Data Availability

The raw data supporting the conclusions of this manuscript will be made available by the authors, without undue reservation, to any qualified researcher. Requests to access the datasets should be directed to judith.streb@uni-ulm.de.
